# Presence of Sildenafil and Tadalafil in Herbal Medicinal Products Claimed to Treat Erectile Dysfunction in Nyamagana and Ilemela Districts, Mwanza, Tanzania

**DOI:** 10.1155/bmri/8222227

**Published:** 2025-07-04

**Authors:** Flora J. Mchallah, Raphael Matinde, Adelina Thomas, Lutugera Kihwili, Tanga Mafuru, James Kapala, Emmanuel Kimaro, Alfredi A. Moyo, Eugene Mutagwaba, Sheila M. Maregesi

**Affiliations:** ^1^Department of Medicinal Chemistry and Pharmacognosy, School of Pharmacy, Catholic University of Health and Allied Sciences, Mwanza, Tanzania; ^2^Department of Pharmaceutics and Pharmacy Practice, School of Pharmacy, Catholic University of Health and Allied Sciences, Mwanza, Tanzania; ^3^Mabibo Research Centre, National Institute for Medical Research, Dar es Salaam, Tanzania

## Abstract

**Background:** Herbal products are increasingly utilized for various conditions, including erectile dysfunction (ED), due to their minimal side effects, affordability, and natural properties. Phosphodiesterase type 5 inhibitors (PDE 5is) such as sildenafil citrate, tadalafil, vardenafil hydrochloride, and avanafil are synthetic oral medications approved for ED treatment. However, multiple studies have highlighted the contamination of herbal aphrodisiacs with PDE 5i or their conventional analogs. This study evaluated the contamination of aphrodisiac-claimed herbal products available in Mwanza, Tanzania.

**Method:** A total of 40 herbal product samples were collected from local vendors and analyzed by high-performance thin layer chromatography (HPTLC). Chromatography was performed on a 20 × 10 cm aluminum-backed plate coated with analytical-grade silica gel 60 F_254_. The plates were developed on a 20 × 20 cm 30-min presaturated twin trough tank containing chloroform (9 mL), methanol (1 mL), and diethylamine (0.1 mL) as the mobile phase. The developed plates were scanned at the wavelengths of 292 and 284 nm for sildenafil and tadalafil, respectively.

**Results:** Out of 40 herbal product samples analyzed, 25 samples (62.5%) were found to be adulterated. Among the adulterated samples, 2 (8%) were contaminated with sildenafil, 9 (36%) with tadalafil, and 14 (56%) with both sildenafil and tadalafil. Quantitative analysis indicated that two samples (12.5%) of the sildenafil-adulterated samples contained an amount of sildenafil that exceeded the maximum recommended daily dose of 100 mg.

**Conclusion:** Aphrodisiac-claimed herbal medicinal products are adulterated with conventional medicines, sildenafil and tadalafil. Alarmingly, some of these products contain higher amounts of these pharmaceutical agents beyond the maximum daily dose.

## 1. Introduction

Herbal medicines are becoming common in low- and middle-income countries (LMICs). Globally, it is estimated that 80% of the population uses herbal medicines to treat illnesses and meet basic medical needs [1]. In Tanzania, 45% of the population still utilizes herbal remedies to treat various health conditions, including erectile dysfunction (ED) [2].

A few side effects, affordability, and natural properties have instigated the increased demand for these products [3]. ED has been a worldwide problem for many years [4]. The prevalence has been shown to range from 3% to 76.5%, with Africa having 53.6%. Studies indicate that the number of men aged 40–70 years affected by ED is expected to double by 2025 in Africa [5–7].

Phosphodiesterase type 5 inhibitors (PDE 5is), mainly sildenafil citrate, tadalafil, vardenafil hydrochloride, and avanafil, are oral synthetic medications approved for treating ED. They work by inhibiting the PDE 5 enzyme in the corpus cavernosum, causing an accumulation of cyclic guanosine monophosphate (cGMP), a molecule that promotes the relaxation of smooth muscle cells and increases blood flow to the penis, resulting in erections [8].

Management of ED by medicinal plants such as ginseng root (*Panax ginseng*), *Mondia whitei*, *Citropsis articulata*, and *Cola acuminata* as well as compounds from natural products such as yohimbine and citrulline has also been testified to improve sexual performance [7]. Herbal aphrodisiacs are particularly popular in African cultures and are preferred to conventional ones. The growing demand for herbal medicines is driven by their minimal side effects, affordability, and natural properties [3]. However, numerous studies have revealed that some of these herbal aphrodisiacs are contaminated with PDE 5i or their conventional substitutes [9].

Recently, in Tanzania, a case was reported in 2022 at Dar es Salaam on the famous herbal product Hensha, commonly known as “*Vumbi la Mkongo*” and supplied by Nyasosi Traditional Clinic, which was banned from the market due to adulteration with sildenafil (Viagra) [10]. Also, a study conducted on a herbal product known as “*AKAYABAGU*” which was banned from the market by the Traditional and Alternative Health Practitioners Council showed that it was adulterated with sildenafil (Viagra, Erecto, or Vega) [11].

Despite existing reports, information on the adulteration of herbal products marketed as aphrodisiacs in Mwanza, Tanzania, remains limited. This study, therefore, seeks to address this gap.

## 2. Materials and Methods

### 2.1. Materials and Reagents

Reference standards of sildenafil citrate and tadalafil active pharmaceutical ingredients were obtained from the Research and Development (R&D) Laboratory of the Muhimbili University of Health and Allied Sciences (MUHAS), using electronic weighing balance, Erlenmeyer volumetric flask, Whatman filter paper (Whatman International Ltd, England), analytical-grade methanol, chloroform, diethylamine, N-hexane, and ethyl acetate, aluminum-backed thin layer chromatography plates 10 × 20 cm normal phase coated with analytical-grade silica gel 60 F_254_ (Merck, Darmstadt, Germany), twin trough development chamber (CAMAG, Muttenz, Switzerland).

### 2.2. Equipment

High-performance thin layer chromatography (HPTLC) consists of TLC Scanner 3 with WinCATS (Version 1.4.3), Linomat 5 semiautomatic applicator (CAMAG, Muttenz, Switzerland) with a Hamilton syringe of 100 *μ* capacity. HPTLC was selected owing to its cost-effectiveness, simplicity, and availability.

### 2.3. Sample Collection

A total of 40 samples of herbal products for ED were randomly purchased from different sellers in Nyamagana and Ilemela districts of Mwanza region in Tanzania based on their predetermined location strata. Each sample was assigned a sample identity (ID), origin, product dosage form, and instructions for use.

After collection, the samples were transferred to the pharmaceutical analysis laboratory and were stored at room temperature in their original packages before analysis.

## 3. Experimental Procedure

### 3.1. Standard Preparation

The procedures were conducted according to the method developed and validated in another study [12]. Sildenafil citrate (100 mg) was weighed and dissolved in 100 mL of methanol to make a standard stock solution of 1 mg/mL concentration. Likewise, 100 mg of tadalafil was weighed and dissolved in 100 mL of methanol to make a stock solution. These solutions were used to prepare calibrators starting from 0.05 to 0.12 mg/mL.

### 3.2. Sample Preparation

For each sample, 10 g was weighed and dissolved in 20 mL of methanol in a volumetric flask. The mixture was filtered, and a portion of 2 mL of the filtrate was taken and methanol was added to 10 mL. From this solution, 1 mL was taken and methanol was added to make 10 mL, and this solution was analyzed.

### 3.3. Chromatographic Procedures

Chromatography was performed on a 20 × 10 cm aluminum-backed plate coated with analytical-grade silica gel 60 F_254_ (Merck, Darmstadt, Germany). Samples (4 *μ*L) were spotted in 5-mm bands on the plate by using a CAMAG Linomat V (CAMAG, Muttenz, Switzerland) sample applicator equipped with a 100-*μ*L syringe.

The plates were developed in a 20 × 20 cm 30-min presaturated twin trough tank containing chloroform (9 mL), methanol (1 mL), and diethylamine (0.1 mL) as the mobile phase according to the method previously developed and validated in another study [13]. The developed plates were studied in the HPTLC Scanner 3 operated using WinCATS Version 1.4.3 at the wavelengths of 292 and 284 nm for sildenafil and tadalafil, respectively.

## 4. Results

**Figure 1:**
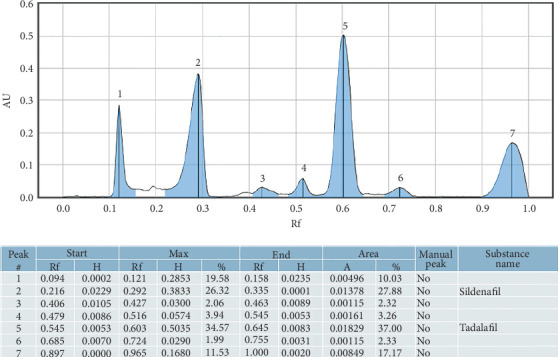
The chromatogram for the reference standard solutions of sildenafil and tadalafil.

**Figure 2:**
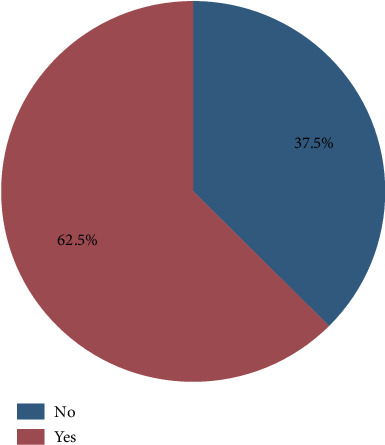
The proportions of adulterated and unadulterated aphrodisiac herbal products.

The retention factors of 0.35 and 0.60 were used as the indicators for sildenafil citrate and tadalafil, respectively, as shown in Figure 1. The findings indicated that of the 40 samples analyzed, 25 (62.5%) were adulterated with one or both of the conventional pharmaceutical compounds, as shown in Figure 2. Among those adulterated, 2 samples (8%) were adulterated with sildenafil alone, 9 samples (36%) were adulterated with tadalafil alone, and 14 samples (56%) were adulterated with both sildenafil and tadalafil (Table 1). Further analysis indicated a 95% confidence interval for proportions was found to be 0.6%–16.92%, 10.84%–38.45%, and 20.63%–51.68% for sildenafil, tadalafil, and both sildenafil and tadalafil, respectively (Table 2).

Quantitative analysis indicated the amount of sildenafil citrate and tadalafil ranged from 3.5 to 146 mg and 0.9–9.5 mg, respectively, as indicated in Table 3.

## 5. Discussion

The study showed that 62.5% of the samples were adulterated with either sildenafil, tadalafil, or both. The magnitude of adulteration in this study was higher compared to a study conducted in Nairobi, Kenya, which was reported to be 32.5% [14]. This discrepancy may be due to the easy accessibility of sildenafil and tadalafil, likely from nonofficial outlets and unscrupulous conventional pharmaceutical dealers. These traditional drugs in herbal medicinal products (HMPs) may suggest a deliberate adulteration to enhance efficacy and increase sales for profit gains.

Another study from Uganda showed 20% adulteration with sildenafil citrate, which is much lower compared to our findings [15]. This could be attributed to a difference in sample collection sites, that is, from registered herbal clinics in Kampala and the control policy which may be different between the two countries.

The sildenafil-adulterated samples were found to contain doses in the range of 3.2–146 mg, subjecting the users unknowingly to amounts beyond the maximum allowable dose, which is 100 mg. These results are comparable to the study done in Kenya, which reported up to 148.7 mg of sildenafil [14]. Nevertheless, these results are contrary to the sildenafil range of 0.45–38.3 mg reported in northwestern Nigeria [16].

Despite these informative findings, our study has some limitations. While HPTLC is cost-effective and suitable for screening, it has lower sensitivity and resolution compared to more advanced techniques like LC-MS or HPLC, potentially leading to false negatives or poor quantification of adulterants. The study's small sample size limits the generalizability of our findings, reducing the ability to make broader conclusions about adulteration trends. Additionally, the study focuses solely on sildenafil and tadalafil, neglecting the possibility of other adulterants such as vardenafil, avanafil, or synthetic analogs, which could also be present in these products. Furthermore, the research covers a limited geographic area, meaning its findings may not reflect the full extent of adulteration in different markets or regions. These limitations highlight the need for more comprehensive studies using advanced analytical methods and larger, more diverse sample sets.

## 6. Conclusion and Recommendation

HMPs used for the treatment of ED are adulterated with conventional medicines, sildenafil and tadalafil. Some of these products contain higher amounts of these pharmaceutical agents beyond the maximum daily dose. Consumption of these products exposes public health to serious risks, and the major concerns are fatal hypotension and priapism, which may lead to permanent penile damage. It is therefore necessary to establish and enforce the strict control of the HMPs. This should be a whistle-blow to responsible regulatory bodies to remain vigilant and stringent. Similarly, governments and health agencies should educate consumers about the risks associated with adulterated herbal aphrodisiacs and how to identify certified, safe products.

We recommend that further studies be conducted on detecting other derivatives of PDE 5i in HMPs and other dosage forms of herbal products with advanced analytical techniques and a large sample size for suitable generalization.

## Figures and Tables

**Table 1:** The proportion of adulterants in adulterated aphrodisiac-claimed herbal products.

**Adulterant**	**Frequency**	**Proportion (%)**
Sildenafil	2	08
Tadalafil	9	36
Sildenafil + tadalafil	14	56
Total	25	100

**Table 2:** The proportions of adulteration in aphrodisiac-claimed herbal products.

**Adulterant**	**Frequency**	**Proportion**	**95% CI**
Sildenafil	2	5%	0.6–16.9
Tadalafil	9	22.5%	10.84–38.4
Sildenafil + tadalafil	14	35%	20.63–51.68
None	15	37.5%	

**Table 3:** Amounts of sildenafil and tadalafil detected in herbal products.

**Sample ID**	**Adulterant**	**Quantity of adulterant**
3504/23	Sildenafil and tadalafil	123 and 0.9 mg
0504/23	Sildenafil and tadalafil	3.5 and 6.4 mg
0704/23	Sildenafil and tadalafil	58 and 2.3 mg
2804/23	Sildenafil	40.6 mg
0804/23	Sildenafil	146 mg
2904/29	Tadalafil	6.5 mg
3604/23	Sildenafil and tadalafil	32.7 and 9.5 mg
4004/23	Tadalafil	6.3 mg

## Data Availability

The data that support the findings of this study are available on request from the corresponding author. The data are not publicly available due to privacy or ethical restrictions.

## Nomenclature

ED: erectile dysfunction
HMPs: herbal medicinal products
HPLC: high-performance liquid chromatography
HPTLC: high-performance thin layer chromatography
LC: liquid chromatography
MS: mass spectrometry
MUHAS: Muhimbili University of Health and Allied Sciences
PDE 5i: phosphodiesterase type 5 inhibitor
TLC: thin layer chromatography
